# Predicting prognosis and immunotherapeutic response of clear cell renal cell carcinoma

**DOI:** 10.3389/fphar.2022.984080

**Published:** 2022-10-14

**Authors:** Jun Wang, Weichao Tu, Jianxin Qiu, Dawei Wang

**Affiliations:** ^1^ Department of Urology, Shanghai General Hospital Affiliated to Shanghai Jiao Tong University School of Medicine, Shanghai, China; ^2^ Department of Urology, Ruijin Hospital, School of Medicine, Shanghai Jiao Tong University, Shanghai, China

**Keywords:** clinical predictive model, kidney cancer, immunotherapy, survival analysis, immune checkpoint inhibitors

## Abstract

Immune checkpoint inhibitors have emerged as a novel therapeutic strategy for many different tumors, including clear cell renal cell carcinoma (ccRCC). However, these drugs are only effective in some ccRCC patients, and can produce a wide range of immune-related adverse reactions. Previous studies have found that ccRCC is different from other tumors, and common biomarkers such as tumor mutational burden, HLA type, and degree of immunological infiltration cannot predict the response of ccRCC to immunotherapy. Therefore, it is necessary to further research and construct corresponding clinical prediction models to predict the efficacy of Immune checkpoint inhibitors. We integrated PBRM1 mutation data, transcriptome data, endogenous retrovirus data, and gene copy number data from 123 patients with advanced ccRCC who participated in prospective clinical trials of PD-1 inhibitors (including CheckMate 009, CheckMate 010, and CheckMate 025 trials). We used AI to optimize mutation data interpretation and established clinical prediction models for survival (for overall survival AUC: 0.931; for progression-free survival AUC: 0.795) and response (ORR AUC: 0.763) to immunotherapy of ccRCC. The models were internally validated by bootstrap. Well-fitted calibration curves were also generated for the nomogram models. Our models showed good performance in predicting survival and response to immunotherapy of ccRCC.

## Introduction

Clear cell renal cell carcinoma (ccRCC) is a common kidney tumor which naturally resistant to chemotherapy and radiotherapy. The immune checkpoint blockade (ICB) has become an essential part of standard care for patients with advanced/metastatic ccRCC. However, only a low proportion of these patients benefit from immunotherapy (Braun et al., 2020). It is critical to identify these appropriate patients who may respond to ICB agents and avoid high medical costs and potential adverse effects for some patients who do not respond to ICBs. Thus, developing clinical predictive models for ICBs efficacy is an urgent unmet medical need.

ccRCC carries a modest tumor mutational burden (TMB) [median of 1.42 mutations per megabase (mut/mb)] (de Velasco et al., 2016), 10-fold lower than melanoma and comparable to immune ‘‘cold’’ tumors (Alexandrov et al., 2013). Unlike other cancer, TMB, human leukocyte antigen (HLA) subtype, and immune cell infiltration does not predict response to ICB agents (Braun et al., 2020; Au et al., 2021). Thus, ccRCC specified investigations are required. PBRM1 truncation mutations have been shown to respond to ICB agents (Miao et al., 2018; Braun et al., 2019). Truncating mutations indicate nonsense mutations, frameshift insertions and deletions, and splice-site mutations. However, In-frame insertions and deletions, missense mutations, and other alterations are not included in the two studies. Chromosomal losses of 9p21.3 and 10q23.31, human endogenous retroviruses (ERVE-4, ERV4700, ERV2282, and ERV3382), transcriptions of ADAMTS14, PARP1, KCNN4, RUFY4, MUC20, LAG3, and PDCD1 were also shown to associate with ICB response (Smith et al., 2019; Braun et al., 2020; Wu et al., 2021; Xue et al., 2022a; Chen et al., 2022b; Cui et al., 2022; Hagiwara et al., 2022; Miao et al., 2022). These biomarkers require further validation in multivariate analyses (Wu et al., 2021).

This study aimed to develop and internally validate models that predict the survival and response of PD-1 agents. In this study, we used deep learning-based MutFormer to stratify missense mutations, In-frame insertions and deletions of PBRM1 (Jiang et al., 2021). We established the clinical predictive models integrating genetic and transcriptomic data from 592 advanced-stage ccRCC patients.

## Materials and methods

### Clinical cohorts

We used the pan-cancer IMPACT2018 dataset described by Samstein et al. (2019). It includes 1,661 patients who represented a variety of cancer types and received immune checkpoint inhibitor (ICI) therapy (atezolizumab, avelumab, durvalumab, ipilimumab, nivolumab, pembrolizumab, or tremelimumab as monotherapy or in combination). Overall survival was measured from the date of first ICI therapy to the event of death or the last follow-up.

Patient data from three clinical trials of the anti-PD-1 agent nivolumab in advanced/metastatic clear cell renal cell carcinoma (ccRCC) were used in this study: CheckMate 009 (CM-009; NCT01358721) (Choueiri et al., 2016), CheckMate 010 (CM-010; NCT01354431) (Motzer et al., 2015) and CheckMate 025 (CM-025; NCT01668784) (Motzer et al., 2018). Corresponding genomic and transcriptomic data were described by Braun et al. (2020).

### MutFormer prediction

MutFormer is a context-dependent transformer-based deep learning model to predict the consequence of missense mutation, in-frame insertions and deletions (Jiang et al., 2021). Mutated sequences were mapped to hg19 genome assembly. Predictions were conducted in a server with AMD EPYC 7502 and GTX3090 GPU. Paired-protein sequences (concatenation of the wildtype and mutated protein sequence of PBRM1) were used to predict the consequence of these mutations.

### Candidate variable selection

The candidate predictors were selected based on literature, clinical importance, the ease of measurement in the real world, and the sample size of the datasets. A complete set analysis was used because missing transcription data can not be imputed using a multiple imputation approach.

### Model build and validation

Multivariate predictive models estimate a patient’s probability that a specific event will occur in the future (prognostic models) or a particular condition is present (such as complete response) based on multiple features for that patient. A backward stepwise approach was applied to multivariate COX or logistic models. Models were compared using Akaike information criterion. Minimum 10 events per effective variables were considered during the building process. The models were internal validation by bootstrap.

### Statistical analysis

Kaplan-Meier survival analysis of progression-free survival (PFS) and overall survival (OS) with hazard ratios (HRs) and 95% confidence intervals (CIs) were stratified by mutation categories or high or low value of integrated PBRM1 score. The cut-off value was defined by the method described by Lausen et al. (2004). Univariate and multivariate Cox regressions were conducted to establish predictive models for OS and PFS. Univariate and multivariate logistic regressions were conducted to establish predictive models for objective response rate (ORR). R 3.6.1 (http://www.r-project.org/) was used for statistical analysis. A *p*-value of < 0.05 was considered statistically significant. Differential gene analysis was performed by package limma 3.42 with a false discovery rate of 0.05 (Ritchie et al., 2015). Kyoto Encyclopedia of Genes and Genomes (KEGG) analysis was performed by package ClusterProfiler 3.14 (Yu et al., 2012).

## Results

### Predicting consequence of PBRM1 mutation by MutFormer

**FIGURE 1:**
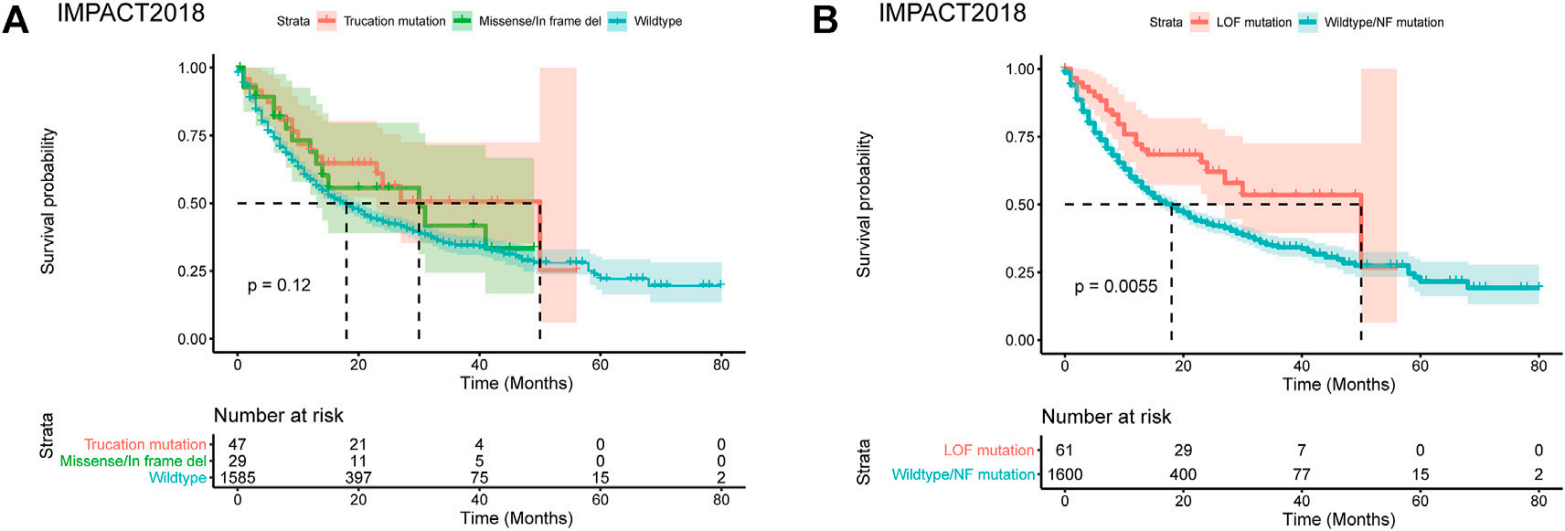
Predicting consequence of PBRM1 mutation by MutFormer. **(A)** The Kaplan-Meier curves of overall survival stratified by truncation mutation, missense/in frame del, wildtype of PBRM1 in the IMPACT2018 datasets. **(B)** The Kaplan-Meier curves of overall survival stratified by loss of function or wildtype/normal function mutation of PBRM1 in the IMPACT2018 datasets. LOF is short for loss of function. NF is short for normal function.

To analyze the role of the missense mutation, in-frame deletions and insertions of PBRM1, we selected a pan-cancer dataset IMPACT2018 with relatively more cases of PBRM1 missense mutation, in-frame deletions (Samstein et al., 2019) (no in-frame insertion in this dataset). This dataset contained the clinical and genomic data of 1,662 advanced cancer patients treated with immune checkpoint inhibitors (ICI). The demographic data has been described by Samstein et al. (2019). As shown in Figure 1A, the curve of the missense mutation, in-frame deletions located between the wildtype PBRM1 and truncation mutations in the IMPACT2018 datasets. The comparison of the three groups was no statistical significance (*p* = 0.12).

Using the MutFormer, we classified the missense mutation, in-frame deletions and insertions cases into the loss of function (LOF) or normal function group. The LOF group significantly had better survival than the normal function group in the IMPACT2018 dataset (*p* = 0.0055, Figure 1B).

### Integrated PBRM1 score outperforms both genomic and transcriptional variables of PBRM1

**FIGURE 2:**
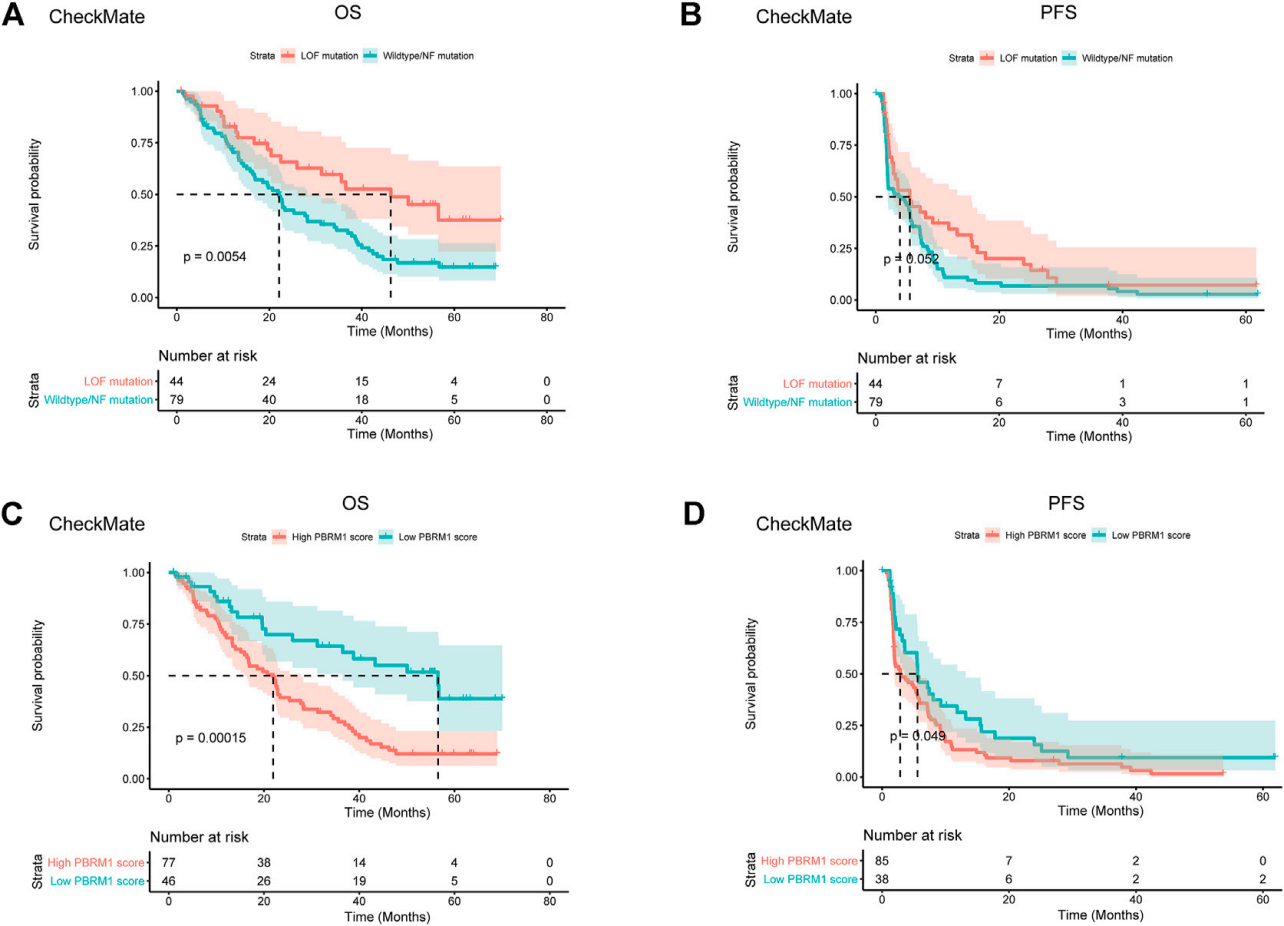
Integrated PBRM1 score outperforms both genomic and transcriptional variable of PBRM1. **(A)** The Kaplan-Meier curves of overall survival stratified by loss of function or wildtype/normal function mutation of PBRM1 in the CheckMate 009, CheckMate 010 and CheckMate 025 datasets. **(B)** The Kaplan-Meier curves of progression free survival stratified by loss of function or wildtype/normal function mutation of PBRM1 in the CheckMate 009, CheckMate 010 and CheckMate 025 datasets. **(C)** The Kaplan-Meier curves of overall survival stratified by high or low integrated PBRM1 score in the CheckMate 009, CheckMate 010 and CheckMate 025 datasets. **(D)** The Kaplan-Meier curves of progression free survival stratified by high or low integrated PBRM1 score in the CheckMate 009, CheckMate 010 and CheckMate 025 datasets. LOF is short for loss of function. NF is short for normal function.

Previous studies have shown that PBRM1’s truncation mutation correlates with the response to immunotherapy, while its transcription does not correlate with the response to immunotherapy (Braun et al., 2020). It is quite reasonable as PBRM1’s truncation mutation affects translation rather than transcription. In monoallelic mutation, theocratically, a half transcript can be translated into functional BAF180 protein. Despite genomic mutation, epigenetic silencing of PBRM1 can also lead to a lack of functional BAF180 protein. We asked whether the integration of both PBRM1’s genomic and transcriptional variables would surpass the PBRM1’s genomic data. We validated this hypothesis in a merged advanced ccRCC dataset with CheckMate 009 (CM-009; NCT01358721), CheckMate 010 (CM-010; NCT01354431) and CheckMate 025 (CM-025; NCT01668784) (Motzer et al., 2018). The demographic data of the merged dataset has been described by Braun et al. (Braun et al., 2020). We built the integrated PBRM1 score with the transcriptional value of PBRM1, reduced the transcriptional value by half if it is a monoallelic mutation and set the transcriptional value to zero if it is a biallelic mutation. The classification of the LOF group and wildtype/normal function group was achieved by MutFormer. The LOF mutations in PBRM1 were associated with improved OS and PFS survival following PD-1 blockade (*p* = 0.0054 and *p* = 0.052) (Figures 2A,B). The integrated PBRM1 score showed a better separation of the high and low PBRM1 score groups for both OS (*p* = 0.00015) and PFS (*p* = 0.049) (Figures 2C,D).

### Univariate analysis of candidate biomarkers

Chromosomal losses of 9p21.3 and 10q23.31, human endogenous retroviruses (ERVE-4, ERV4700, ERV2282, ERV3382), transcriptions of ADAMTS14, PARP1, KCNN4, RUFY4, MUC20, LAG3 and PDCD1 which previously shown to associate with ICB response were included in the univariate analysis (Smith et al., 2019; Braun et al., 2020; Wu et al., 2021; Xue et al., 2022a; Chen et al., 2022b; Cui et al., 2022; Hagiwara et al., 2022; Miao et al., 2022). Transcriptions of CDKN2A, CDKN2B, CDKN2B_AS1, and MTAP were included because these genes are located at 9p21.3, and they are also potential tumor suppressors (Girgis et al., 2012). TKT was included because it is located close to 9p21.3.

**FIGURE 3:**
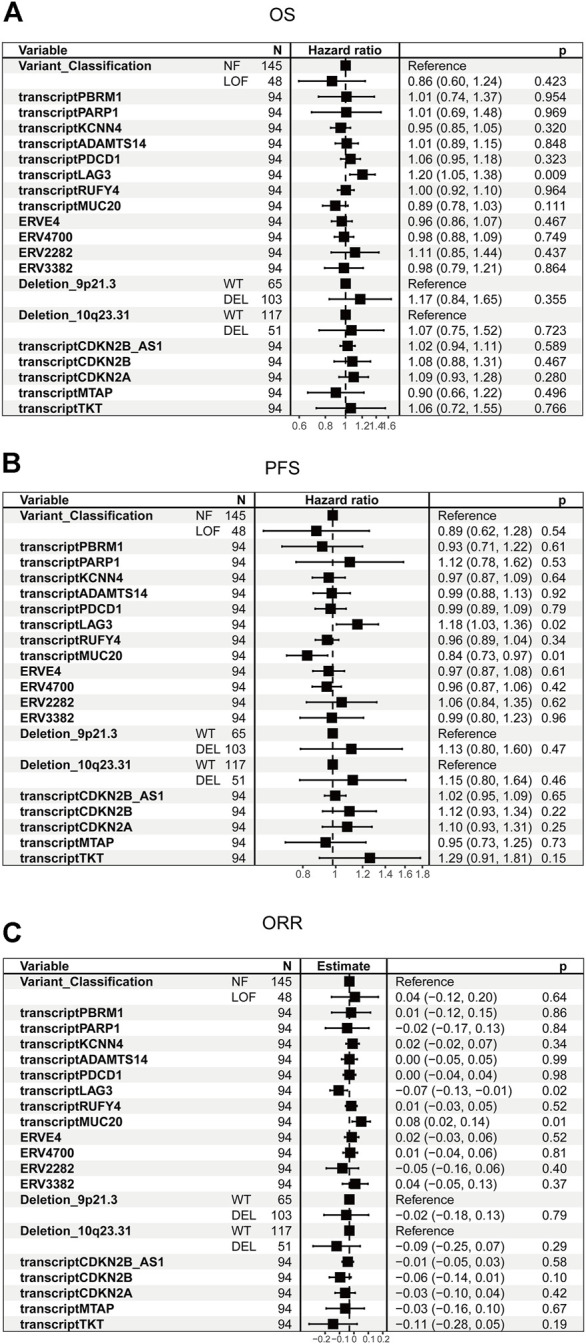
Univariate analysis of candidate biomarkers in the control cohort. **(A)** The univariate analysis based on Cox regression was used to assess the correlation between OS and candidate biomarkers in the control cohort. **(B)** The univariate analysis based on Cox regression was used to assess the correlation between PFS and candidate biomarkers in the control cohort. **(C)** The univariate analysis based on logistic regression was used to assess the correlation between ORR and candidate biomarkers in the control cohort.

The univariate cox analysis in control groups in which patients were treated with everolimus showed that LAG3 was associated with OS (*p* = 0.009) and PFS (*p* = 0.02) survival (Figures 3A,B). MUC20 was also associated with PFS (*p* = 0.01) survival (Figure 3B). Similar results can be found in Figure 3C. These results indicated that LAG3 and MUC20 were survival predictors rather than predictors for immunotherapy efficacy.

**FIGURE 4:**
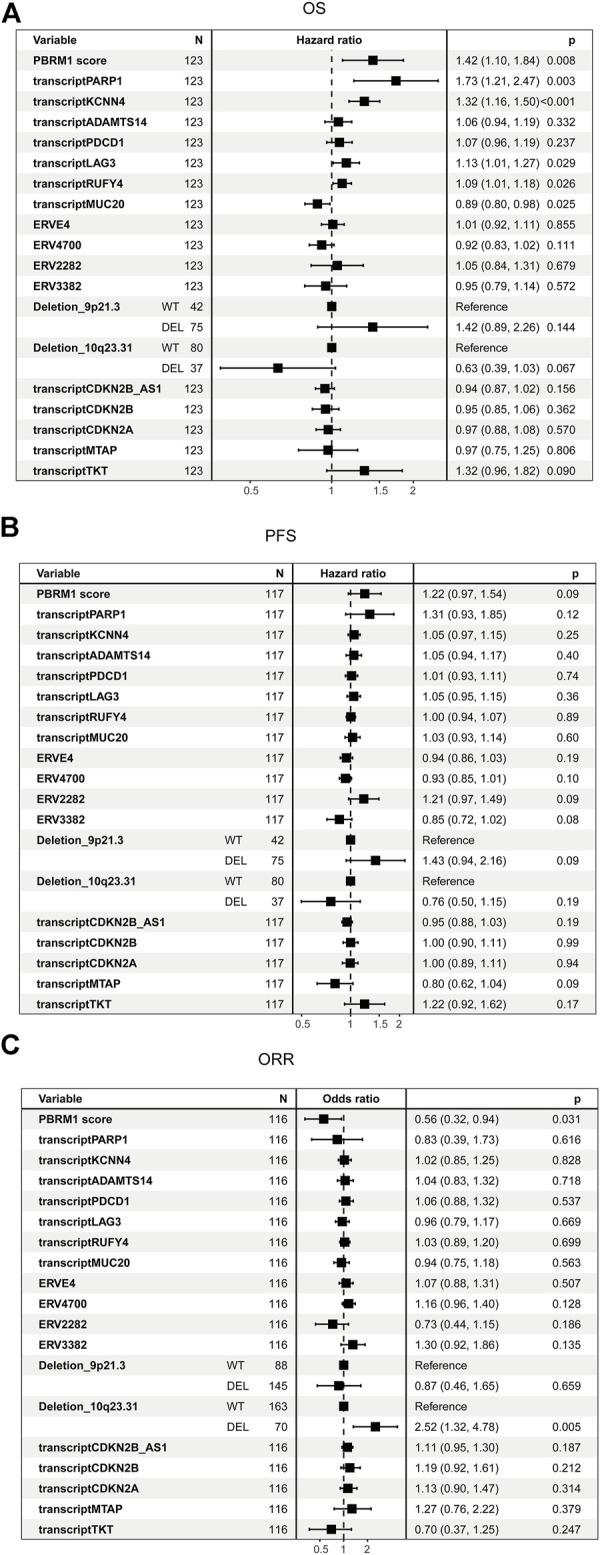
Univariate analysis of candidate biomarkers in the PD-1 cohort. **(A)** The univariate analysis based on Cox regression was used to assess the correlation between OS and candidate biomarkers in the PD-1 cohort. **(B)** The univariate analysis based on Cox regression was used to assess the correlation between PFS and candidate biomarkers in the PD-1 cohort. **(C)** The univariate analysis based on logistic regression was used to assess the correlation between ORR and candidate biomarkers in the PD-1 cohort.

The univariate cox analysis in PD-1 groups showed that integrated PBRM1 score (*p* = 0.008), transcription of PARP1 (*p* = 0.003), KCNN4 (*p* < 0.001), RUFY4 (*p* = 0.026), deletion of 10q23.31 were associated with OS (Figure 4A). The correlations between integrated PBRM1 score, ERV2282, deletion of 9p21.3, transcription of MTAP, and PFS have the lowest *p* values (*p* = 0.09) among all analyzed variables (Figure 4B). None of these variables had a *p* value < 0.05, maybe due to the small sample size. The integrated PBRM1 score (*p* = 0.031) and deletion of 10q23.31 (*p* = 0.005) were associated with ORR in the univariate logistic analysis (Figure 4C).

### Multivariate predictive models

**FIGURE 5:**
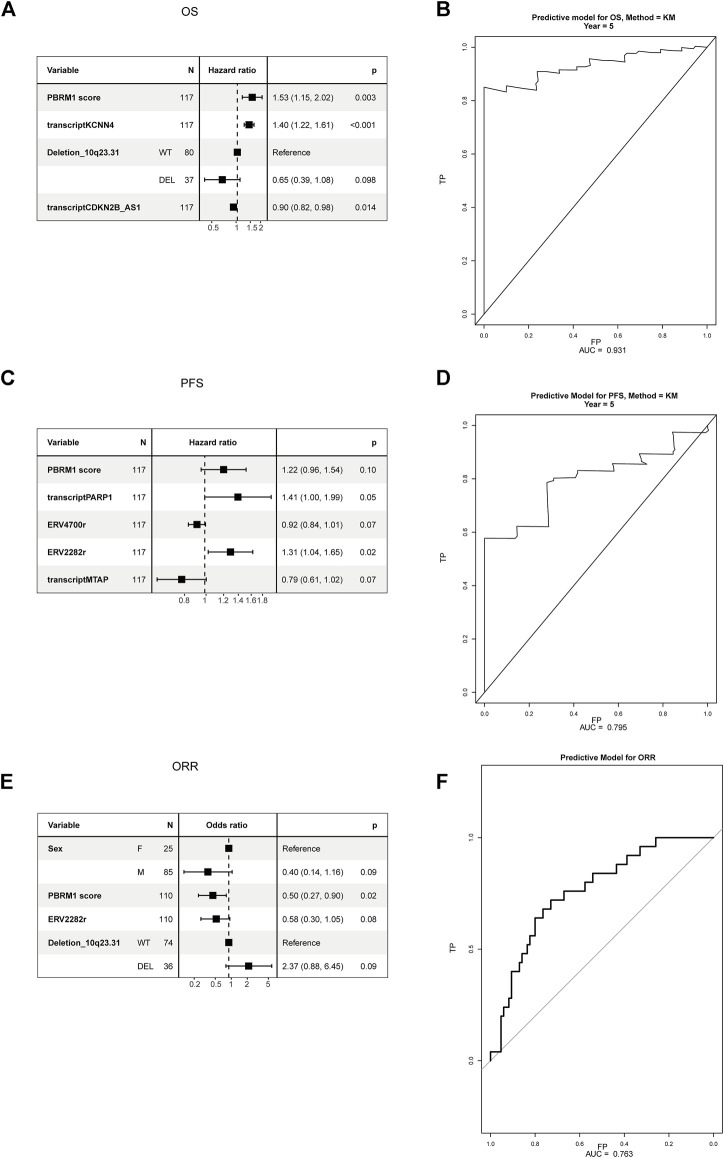
Multivariate analysis of candidate biomarkers in the PD-1 cohort. **(A)** The multivariate analysis based on Cox regression was used to assess the correlation between OS and candidate biomarkers in the PD-1 cohort. **(B)** The ROC curve of predictive model of OS. **(C)** The multivariate analysis based on Cox regression was used to assess the correlation between PFS and candidate biomarkers in the PD-1 cohort. **(D)** The ROC curve of predictive model of PFS. **(E)** The multivariate analysis based on logistic regression was used to assess the correlation between ORR and candidate biomarkers in the PD-1 cohort. **(F)** The ROC curve of predictive model of ORR.

All candidate biomarkers with *p* values < 0.2 in the univariate analysis were included in the multivariate analysis. Variable selection was achieved by backward stepwise selection. The predictive COX model for OS was built and included integrated PBRM1 score, KCNN4, deletion of 10q23.31, and CDKN2B_AS1, with an area under the curve (AUC) 5 0.931 (Figures 5A,B). The predictive COX model for PFS was built and included an integrated PBRM1 score, transcript of PARP1 and MTAP, ERV4700, and ERV2282, with an AUC of 0.795 (Figures 5C,D). The predictive logistic model for ORR was built and included integrated PBRM1 score, sex, ERV2282, and deletion of 10q23.31, with an AUC 5 0.763 (95% 0.661–0.864) (Figures 5E,F). The predictive models were internally validated by bootstrap. The bootstrap validating c-index for the prediction of OS was 0.700 (95% CI 0.68–0.707), close to the training c-index of 0.712 (95% CI 0.653–0.770). The bootstrap validating c-index for the prediction of PFS was 0.603 (95% CI 0.575–0.620), close to the training c-index of 0.631 (95% CI 0.564–0.685).

### Calibration and nomogram

**FIGURE 6:**
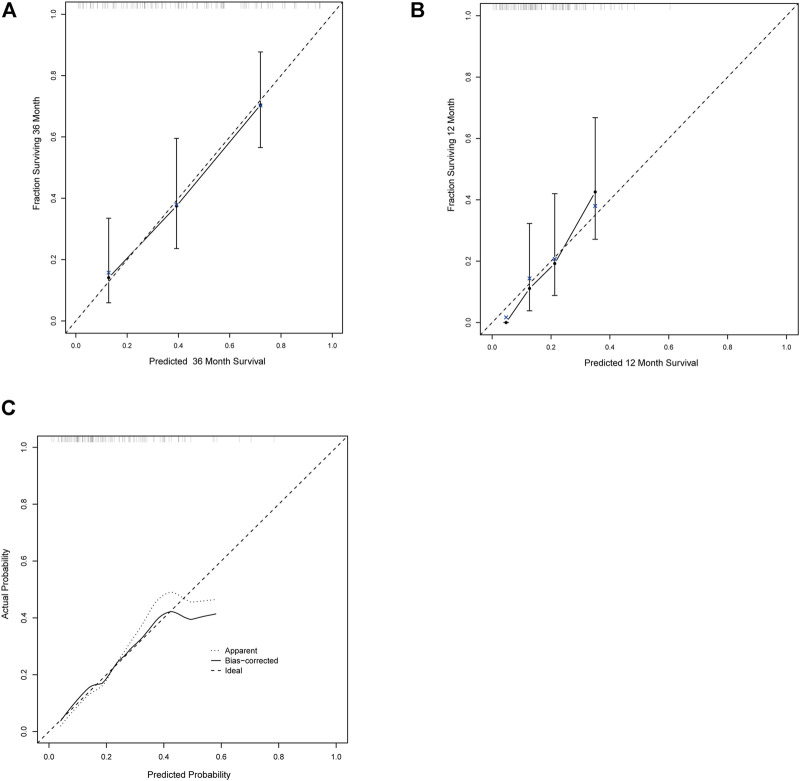
The calibration curves for the probability of OS, PFS, and ORR. **(A–C)** The calibration curves for the probability of OS **(A)**, PFS **(B)**, and ORR **(C)**.

**FIGURE 7:**
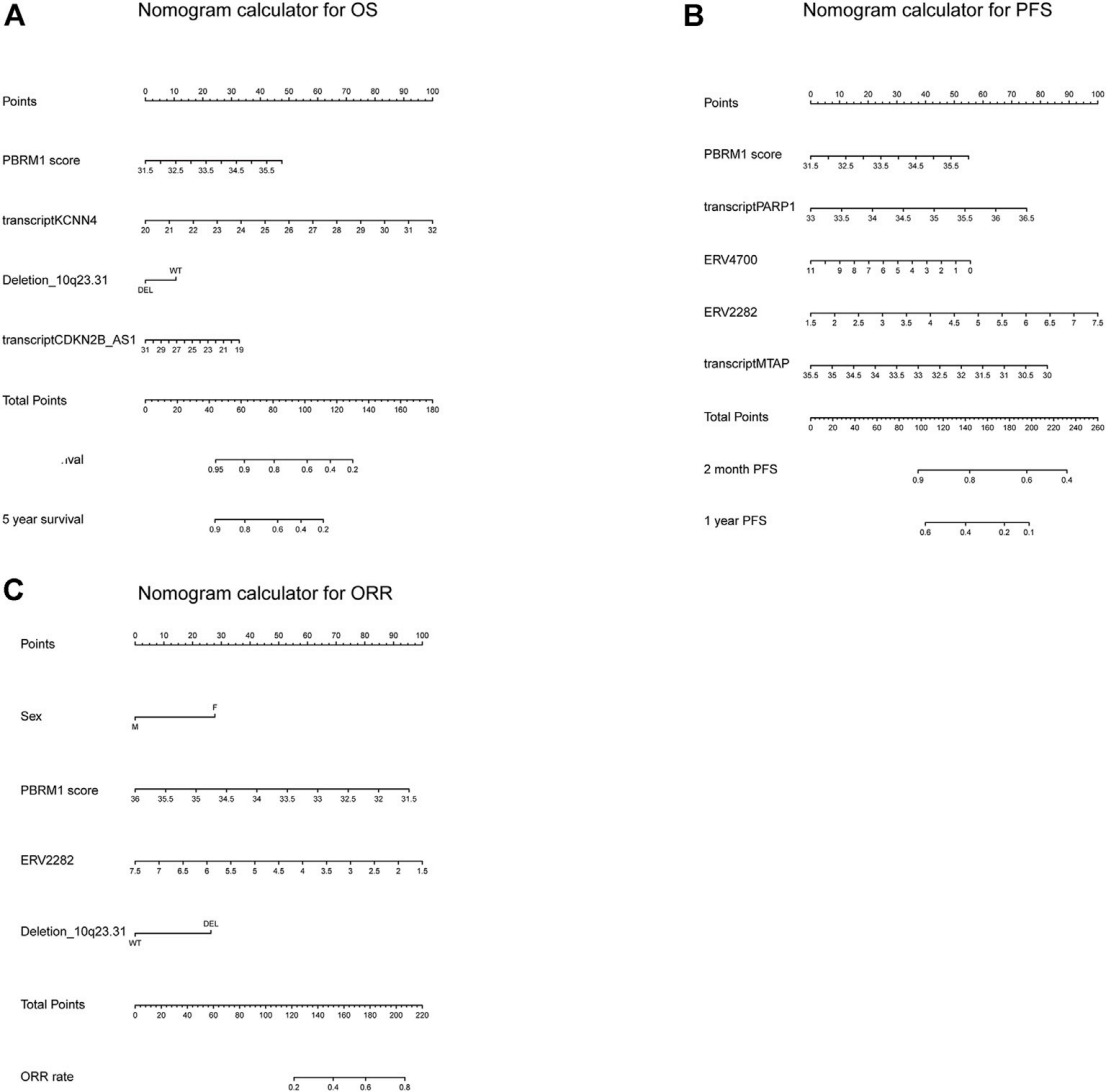
The nomogram calculator for OS, PFS, and ORR. **(A–C)** The nomogram calculator for OS **(A)**, PFS **(B)**, and ORR **(C)**.

The calibration curves for the probability of OS, PFS, and ORR showed that the prediction models had good consistency with the actual observation (Figure 6). To visualize the predictive models, nomograms were generated to facilitate the prediction of OS, PFS, and ORR (Figure 7).

### Differential genes and pathways between high and low-risk groups

We attempted to explore the transcriptomic differences between high and low risk groups of the OS predictive model. We found that KCNN4 is the top differential genes. All the differential genes were demonstrated in Supplementary Table S1. KEGG pathway analysis indicated that the differentially expressed genes were mostly enriched in amino acid metabolism, fatty acid degradation, and HIF−1 signaling pathway (Supplementary Figure S1).

## Discussion

With the advent of immunotherapy, we already target some of the immune system pathways in ccRCC, but ICIs are only effective in some ccRCC patients. Thus, it would be important to predict prognosis/response for specified patients. New prognostic and responsive biomarkers and integrative predictive models are required for clinical practices. We developed nomograms that will be clinically useful and forecast clinical outcomes from checkpoint inhibitor immunotherapy in advanced ccRCC. Compared with these previous researches, we used the real clinical outcomes and survival data from patients with anti-PD1 immunotherapy, which are more reasonable and reliable. Xie et al. (2021) and Chen et al. (2021) established and validated predictive models with a panel of immune-related genes. However, they predicted the survival of all ccRCC patients, even though these patients may not receive immunotherapy at all (Chen et al., 2021; Xie et al., 2021). Gao et al. (2021) trained predictive models with a dependent variable which were predicted using machine learning algorithms rather than real patient responses (Charoentong et al., 2017). Furthermore, we found that LAG3 and MUC20 were associated with prognosis in the control group that did not receive immunotherapy, which indicated that LAG3 and MUC20 are universal prognosis predictors rather than predictors for immunotherapy.

Another prognostic model MSKCC was developed based on patients’ data between 1975 and 1996 when PD-1 therapy has not been developed (Motzer et al., 1999). It includes Karnofsky performance status, lactate dehydrogenase, serum albumin, corrected serum calcium, and time from diagnosis to systemic treatment (Motzer et al., 1999). However, this model has not been validated in the modern era of ICI. No evidence has shown it predict response to immunotherapy. In addition, MSKCC tend to categorize the bulk of the patients into the intermediate-risk category. Revised models that consider the response to immunotherapy are needed.

The adverse effect of immunotherapy included colitis, diarrhea, nausea, vomiting, headache, itching, skin photosensitivity and arthritis-type pain (Brahmer et al., 2021). With the predictive models, we can filter unresponsive patients and avoid these patients exposed to the potential risk of ICIs. Apart from that, developing and validating predictive biomarkers for the early identification of at-risk patients are also another important area of research (Brahmer et al., 2021). The current predictive models were mainly trained and internally validated with patients who use ICIs as second-line treatment. The prediction efficacy of the models in patients using first-line ICIs or combined treatment was still unclear. Further research is required.

As a functional PBRM1 interacted with other components of the PRAF complex with its c-terminal, a truncation mutation theoretically always leads to PBRM1 loss of function (Gao et al., 2017). However, a point missense mutation or an in-frame deletion or insertion does not always cause functional alterations (Gao et al., 2017). We used the deep learning-based model MutFormer which receives long protein sequence inputs and make predictions based on contexts. While some tools only work for missense mutations (Jordan et al., 2011; Tang and Thomas, 2016). With MutFormer, we could analyze the consequence of all categories of mutations, including nonsense mutation, frame shift mutation, missense mutation, in-frame deletion and insertion.

PBRM1 was presented in each predictive model. PARP1 and MTAP were presented only in the predictive model for PFS. Poly (ADP-ribose) polymerase 1 (PARP1) is an ADP-ribosylating enzyme that plays roles in DNA repair, maintenance of genomic integrity, and transcriptional regulation (Caldecott, 2022). More interestingly, the interplay between PARP1 and PBRM1 has been found (Lanillos et al., 2022). *In vivo* studies have shown that PARP1 and ATR inhibition are synthetic lethal with PBRM1 defects (Chabanon et al., 2021). However, further investigation is required for PARP1’s effect on ccRCC treated with tyrosine kinase inhibitors required further investigation. PARP1 is associated with PFS in ccRCC treated with sunitinib. In summary, PARP1 can be not only a predictor but also a potential treatment target for ccRCC. Methylthioadenosine phosphorylase (MTAP) is a key enzyme in the methionine salvage pathway, responsible for regenerating methionine and adenine (Marjon et al., 2016). MTAP frequently becomes deficient in cancer and reprograms the metabolism by building up methylthioadenosine. Unlike the tumor suppressor role reported by other literature, in the both univariate and multivariate COX analysis for PFS and univariate logistic analysis for ORR, higher MTAP expression was associated with shorter PFS and lower ORR, which is consistent with the results reported by Alhalabi et al. (2021). Accumulating methylthioadenosine could inhibit the methylation of STAT1, leading inhibition of interferon signaling pathways (Mowen et al., 2001). Defects in the interferon pathway may compromise antitumor immune responses (Gao et al., 2016). LncRNA CDKN2B_AS1 is associated with the prognosis of thyroid cancer and the status of the immune microenvironment (Xue et al., 2022b). It also interacts with mitogen-activated protein kinase (MAPK) inactivator dual-specificity phosphatase 1 (DUSP1) and inhibits DUSP1’s activity (Pan et al., 2021). KCNN4 was a potentially heterotetrameric voltage-independent potassium channel that is activated by intracellular calcium in T-lymphocytes. It was selected as one of the variables for the OS predictive model. It was also differentially expressed in the low and high-risk groups. KCNN4 expression was correlated to tumor mutational burden and microsatellite instability levels in 14 types and 12 types of pan-cancers (Chen et al., 2022a). KCNN4 influences tumor microenvironment by remodeling tumor-infiltrating immune cell profiles (Chen et al., 2022a). It may regulate immune response *via* raising Tregs and diminishing resting mast cells in ccRCC, which may cause the differential clinical outcomes (Cui et al., 2022).

More interestingly, multiple metabolism pathways, especially various amino acid metabolism pathways, were enriched in the KEGG analysis. Tryptophan metabolism has been extensively investigated for its immunosuppressive role in the tumor microenvironment (Zhang et al., 2022). Leucine is a nutrient signal that activates complex 1 of the mammalian target of rapamycin (mTORC1), which is a critical regulator of T cell proliferation, differentiation, and function (Ananieva et al., 2016). Furthermore, arginine could be important to T-cell activation and thus modulate innate and adaptive immunity to promote tumor survival and growth (Kim et al., 2018). In summary, multiple amino acid metabolism pathways were enriched in ccRCC, which is consistent with that ccRCC is generally considered a metabolic disease (Qi et al., 2021).

Our study had several limitations. First, although we collected data from three large prospective clinical trials, a proportion of patients had missing genomic and transcriptomic data. We used a complete set analysis which can cause bias. The effective sample size is small. Multiple imputation tests might not be appropriate for transcription data. Second, the clinical predictive models required external validations in new datasets.

## Data Availability

The original contributions presented in the study are included in the article/Supplementary Material, further inquiries can be directed to the corresponding authors.

## Supplementary material

SUPPLEMENTARY FIGURE S1 The pathway enrichment analysis. KEGG enrichment analysis for differential expressed gene.
