# Supplementary figures and images for: Predicting prognosis and immunotherapeutic response of clear cell renal cell carcinoma
Source: Front Pharmacol. 2022 Oct 14;13:984080. doi: 10.3389/fphar.2022.984080 (PMC9614164; doi:10.3389/fphar.2022.984080)

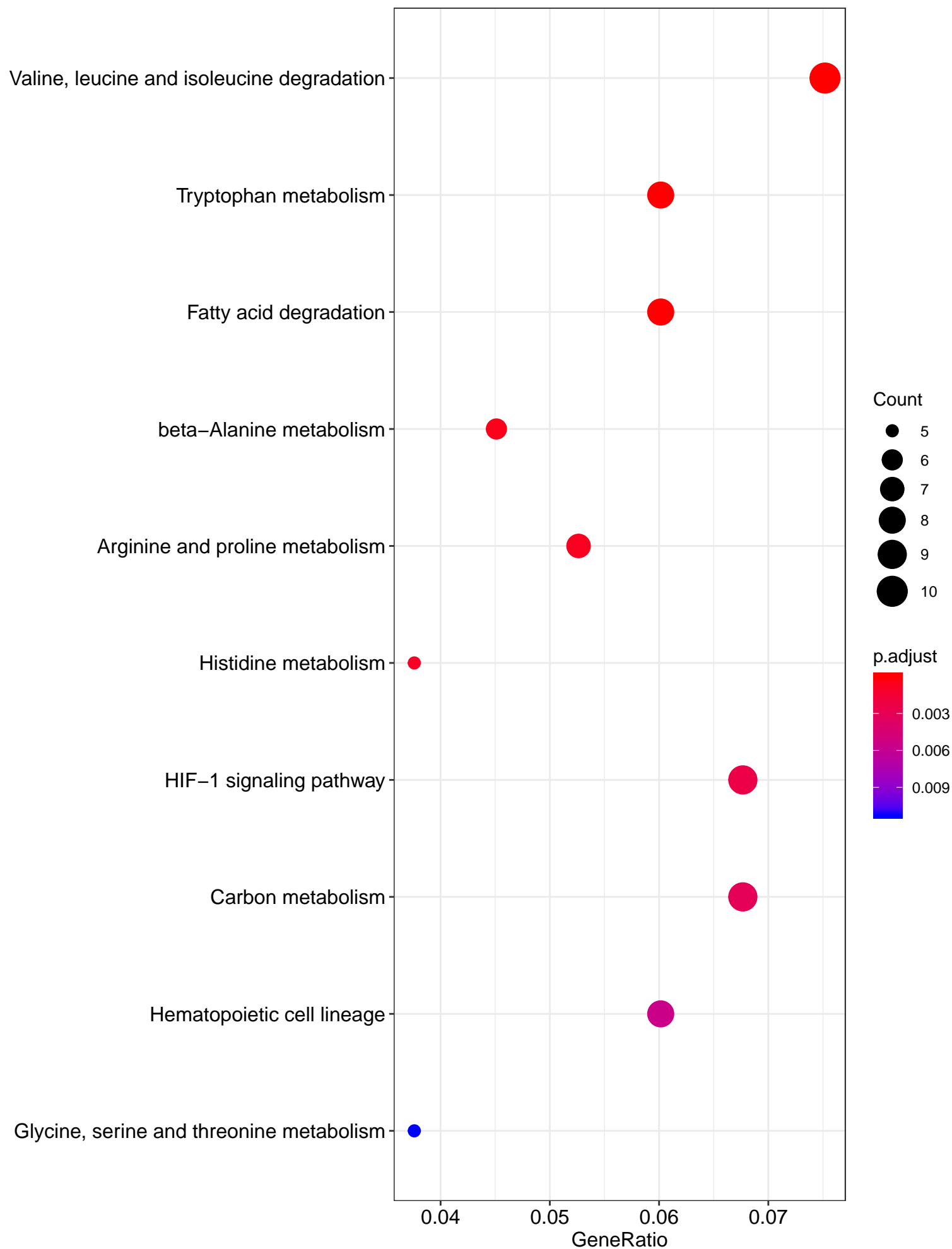

Supplement: Supplementary file 2 [file Image1.pdf]
